# Advertisement

**Published:** 1857-07

**Authors:** 


*Advertisement.*

We would invite the attention of our readers to the advertisement of Messrs. Sargent & Ilsley, druggists, of this city. We are well acquainted with them, and have the fullest confidence in their integrity and skill in the selection and preparation of medicines.

